# Case in Practice

**Published:** 1875-07

**Authors:** B. F. Farley

**Affiliations:** Chebanse, Ill.


Article II.

### CASE IN PRACTICE.

By B. F. FARLEY, M.D., Chebanse, Ill.

Mr. C., bachelor, æt. 62, by occupation a farmer. This patient consulted me in the early part of March last, at which time I gathered the following history: About six weeks previously he had been attacked with very severe diarrhœa, with a moderate amount of pain and tenderness, distressing nausea without emesis, thirst, more marked early in the disease. The discharges from the beginning have been simply "frequent, thin and watery."

The patient had been under treatment since the first symptoms of a serious nature made their appearance. He now complains most of debility, pain and tenderness in the hypogastrium and left hypochondrium, also pain in umbilical and either lumbar region, but less marked than in the hypogastric. The tongue is slightly coated whitish, the margin and tip being abnormally red.

There has been much headache, and vertigo on assuming a sitting or standing posture; the appetite is variable, and the ingestion of food produces pain and flatulency, followed, in a short time, by one to three passages in quick succession; the pulse is rather frequent and small.

I prescribed hyd. chlo. mit., rhei. pul., and sodæ bicarb., in moderate quantities, once in three hours, until very free catharsis was produced, followed by quinia sul., bismuth sub nit., tannin, and opium, two to five times a day, to control diarrhœa, and directed him to return in about one week.

Upon the patient's second visit, he complained of feeling somewhat better but not free from diarrhœa; there was less pain and tenderness than on his first visit. Now I have come to the remarkable feature of this case: the passage, by the patient, of a number of pieces of *oyster shells*, very irregular in form, and in size varying from two or three lines to one inch in diameter; the larger piece is not less than one inch in its shortest diameter. These bodies produced, in their passage, some hæmorrhage, but not very considerable; the shells were passed during the catharsis produced by the calomel and rhubarb. During the few days following, large amounts of the mucous membrane sloughed and came away with the passages from the bowels, and have continued to appear, from time to time, in large amounts. The patient tells me that many times he has been obliged to remove the accumulations with his fingers, the efforts of defecation not being sufficient for their expulsion. The amount of membrane which has been passed, and which I have seen is truly remarkable, and although I saw neither the shell nor membrane *when* passed, I have seen the latter in the fecal matter *as* passed, and have every reason to believe the statements of the patient, especially as he is a man of much intelligence, and truthful.

The treatment has been tonic and astringent, principally, such as a pill of opium and argenti nit., powders of opium, quinine, and bismuth, and such like treatment, with carefully regulated diet.

The patient is now improving gradually. It is the rarity of such cases that has induced me to write this up thus hastily, as I have done. If I have reported anything interesting or instructive, I have my reward.

---

ARTICLE III.

